# *SNCA* Gene, but Not *MAPT*, Influences Onset Age of Parkinson's Disease in Chinese and Australians

**DOI:** 10.1155/2015/135674

**Published:** 2015-04-15

**Authors:** Yue Huang, Gang Wang, Dominic Rowe, Ying Wang, John B. J. Kwok, Qin Xiao, Frank Mastaglia, Jun Liu, Sheng-Di Chen, Glenda Halliday

**Affiliations:** ^1^Neuroscience Research Australia and The University of New South Wales, Sydney, NSW 2031, Australia; ^2^Department of Neurology and Institute of Neurology, Ruijin Hospital Affiliated to Shanghai Jiaotong University School of Medicine, Shanghai 200025, China; ^3^Australian School of Advanced Medicine, Macquarie University, Sydney, NSW 2109, Australia; ^4^Institute of Immunology & Infectious Diseases, Murdoch University, Perth, WA 6150, Australia

## Abstract

*Background*. *α*-Synuclein (*SNCA*) and microtubule-associated protein tau (*MAPT*) are the two major genes independently, but not jointly, associated with susceptibility for Parkinson's disease (PD). The *SNCA* gene has recently been identified as a major modifier of age of PD onset. Whether *MAPT* gene synergistically influences age of onset of PD is unknown. *Objective*. To investigate independent and joint effects of *MAPT* and *SNCA* on PD onset age. *Methods*. 412 patients with PD were recruited from the Australian PD Research Network (123) and the Neurology Department, Ruijin Hospital Affiliated to Shanghai Jiaotong University, China (289). *MAPT* (rs17650901) tagging H1/H2 haplotype and *SNCA* (Rep1) were genotyped in the Australian cohort, and *MAPT* (rs242557, rs3744456) and *SNCA* (rs11931074, rs894278) were genotyped in the Chinese cohort. SPSS regression analysis was used to test genetic effects on age at onset of PD in each cohort. *Results*. *SNCA* polymorphisms associated with the onset age of PD in both populations. *MAPT* polymorphisms did not enhance such association in either entire cohort. *Conclusion*. This study suggests that, in both ethnic groups, *SNCA* gene variants influence the age at onset of PD and *α*-synuclein plays a key role in the disease course of PD.

## 1. Introduction

Parkinson's disease (PD) is the most common neurodegenerative movement disorder in the elderly (approximately 2% of the population aged over 60) with an average age of onset of 60 years and a variety of different subtypes [1, 2]. Patients with young disease onset often have a benign disease course and a lower rate of dementia compared to those with later disease onset [3], and previous studies show that genetic factors influence both the age of onset [4, 5] and clinical subtypes of PD [6–8]. These clinical variations are not due to mutations in PD causative genes [9].

The two most consistently identified susceptibility genes for sporadic PD are the *α*-synuclein (*SNCA*) and microtubule-associated protein tau (*MAPT*) genes [10, 11] which play independent, but not joint, effects on the risk of developing PD [12, 13], although there are significant differences in the variants associated with PD between Asian and Caucasian populations [14]. In addition, we have shown that in Caucasians the* SNCA* and* MAPT* genes act synergistically to influence the rate of progression of PD (certain genotypes have a 5.8-increased risk for developing a more rapid disease progression) when analysing one microsatellite (Rep1 or D4S3481 with three common alleles, that is, 259 bp, 261 bp, and 263 bp or alleles 0, 1, and 2) marker of* SNCA* gene and a rs17650901 SNP of* MAPT* gene (lies in exon 1 and its A-allele tags the* MAPT *H1 haplotype [15]) in an Australian cohort [7]. A recent study showed that among 17 genome-wide association studies- (GWAS-) linked loci in mainland China, only two SNPs (rs11931074 and rs894278) of the* SNCA* gene associate with the risk for sporadic PD after adjusting for age and sex [16]. The rs894278 SNP is located in intron 4, and the rs11931074 remains distal to the untranslated region of* SNCA* [17]. The* MAPT* gene does not appear to be associated with PD susceptibility in the Chinese [16], possibly due to ethnicity and the extremely low frequency of the H2* MAPT *haplotype in mainland China [18]. However, the* MAPT *H1c subhaplotype (tagged by the rs242557 A-allele [19]) and other SNPs (e.g., rs3744456) are associated with increased expression of tau (especially four repeat transcripts) in human brain tissue [20, 21] and* in vivo* experiments [22]. These different* MAPT* SNPs might be associated with PD risk in the Chinese.

It has recently been shown that the* SNCA* gene is a major modifier of age of PD onset [23]. However it remains unclear whether the* MAPT* gene also modified age of PD onset and whether there is a synergistic effect of both* SNCA* and* MAPT* on the age of onset of PD. This study is to investigate independent and joint effects of* MAPT* and* SNCA* on PD onset age. Understanding the influence of variations in these genes on clinical features of PD in different ethnic populations would further consolidate the molecular pathophysiologic mechanisms underpinning PD.

## 2. Methods

### 2.1. Study Subjects

412 patients satisfying the Queen Square Brain Bank Criteria for PD and without autosomal dominant family history of PD were recruited consecutively from the Australian Parkinson's Disease Research Network, Australia (Caucasian: *n* = 123, 66 male, 57 female) and the movement disorders clinic, Department of Neurology, Ruijin Hospital Affiliated to School of Medicine, Shanghai Jiaotong University, China (Han: *n* = 289, 170 male, 119 female) (Table 1). The average age at recruitment (±standard deviation) was 68 ± 9.0 years in Australia and 63 ± 9.4 years in China. The studies were approved by the appropriate institutional ethics committees of the University of New South Wales and the School of Medicine, Shanghai Jiaotong University. Blood from each patient was taken with consent for genetic analyses. Genomic DNA was extracted from peripheral blood by a standardized phenol/chloroform extraction method.

### 2.2. Clinical Information

At recruitment, a standard questionnaire was completed to obtain detailed clinical information, such as gender, age at onset, age at enrolment, medication administration, and family history. The age of onset of PD was defined when at least two of the three main signs of PD, that is, rigidity, tremor, and bradykinesia, had developed [24]. The average age at onset (± standard deviation) was 60 ± 11 years in the Australian cohort (range 32–83 years) and 58 ± 10 years in the Chinese cohort (range 34–82 years).

### 2.3. Genetic Analysis

Due to population-specific heterogeneity in PD [25],* MAPT* (rs17650901) and* SNCA* Rep1 (D4S3481) were genotyped in the Australian cohort [7, 15, 26], and* MAPT* (rs2425577 and rs3744456) and* SNCA* (rs11931074, rs894278) were genotyped in the Chinese cohort [22] (see supplementary Table  1 in supplementary material available online at http://dx.doi.org/10.1155/2015/135674). The rate of genotype calls was ≥95% for all SNPs. For those variants identified by restriction fragment length polymorphism (RFLP), the genotype results were further confirmed via direct PCR product sequencing on random samples. An online tool (http://www.genes.org.uk/software/cubex/) [27] was used to assess linkage disequilibrium in the selected SNPs.

### 2.4. Statistical Analyses

Different models of inheritance were adopted for analysing each polymorphic effect on age at PD onset using one-way ANOVA, where onset age was considered as a continuous variable. As more males have PD, SPSS regression analyses were then used adjusting for gender to minimize this effect. After examining the effects of single polymorphisms on onset age in all subjects, gene-gene interactions were assessed in each cohort using adjusted regression models, and a *P* value of <0.05 was considered as significant.

## 3. Results

Our results showed that the* SNCA* gene, but not the* MAPT* gene, associated with age of PD onset in the cohorts assessed. No synergic genetic effects were detected on age of PD onset between* SNCA *and* MAPT* polymorphisms in either the Australian or Chinese cohort. There was a weak linkage disequilibrium between* SNCA* rs11931074 and rs894278 (*D*′ = 0.72) and there was no linkage disequilibrium between* MAPT* rs2425577 and rs3744456 (*D*′ = 0.44) in the Chinese cohort.

### 3.1. *SNCA*, but Not* MAPT* Gene, Associates with Age of PD Onset

SPSS-ANOVA analysis showed that polymorphisms in* SNCA* only, but not* MAPT* gene, associated with the age of onset of PD in both populations sampled (Table 2). In the Australian cohort, the genotype of* SNCA* D4S3481 associated with onset age of PD. The genetic associations were consistent with recessive models of inheritance of* SNCA* D4S3481 allele 1, although dominant and additive models of allele 0 and allele 1 also had significant effects on the age of PD onset (Table 2). In the Chinese cohort, only* SNCA* rs894278 SNP associated with PD onset age, which followed a recessive inheritance model for allele G.

### 3.2. Genetic Effects of* SNCA* Gene on Age of Onset of PD

After adjusting for gender, SPSS regression analysis showed that* SNCA* polymorphisms were still associated with the onset age of PD, although the effect observed for the* SNCA* D4S3481 allele 1 in Australians is more obvious compared with the* SNCA* rs894278 SNP in the Chinese. Australians carrying two* SNCA* D4S3481 allele 1 had a delayed onset of PD by about seven years (*P* = 0.002), while the Chinese with a* SNCA* rs894278 GG genotype had an earlier onset by about three years (*P* = 0.015) (Table 3 and supplementary figure).

## 4. Discussion

Whether a person might develop PD (susceptibility of PD) and when a patient with PD starts to show the symptoms (PD onset) are two distinct questions. It is not surprising that the data derived from two distinct ethnic cohorts show that polymorphisms in the* SNCA* gene can influence the age of PD onset, while polymorphisms in the* MAPT* gene do not, although* MAPT* gene has been shown directly or indirectly (by regulating other PD risk genes) to be associated with PD in both populations [28–30].

Our data showing that the* SNCA* gene affects age of PD onset in Australian and Chinese cohorts is consistent with a recent report using a very large sample cohort [23] and also with other similarly sized population studies in Spain [31], Germany [23], the UK [13], and Greece [32]. The effect of the* SNCA* gene on age of PD onset is even observed in patients carrying leucine rich repeat kinase 2 (LRRK2) gene mutations [33]. There is stronger* SNCA* gene effects on PD onset age in the Australians compared to that in the Chinese, possibly due to the testing of different polymorphisms, as previously shown [34, 35]. In different populations, the same polymorphism of* SNCA* seems to have variable strengths of effects on PD onset [36], possibly due to other modifiers.

Identifying genes associating with onset of PD has potential for therapeutic targeting. If interventions could delay the onset of symptoms, for some this may effectively “cure” their disease by delaying symptom onset to beyond their life span, while for others it would significantly reduce morbidity and enhance the quality and productivity of their life.

Expression data show that compared to* SNCA* “protective” alleles D4S3481 allele 0 (259 bp) [37] and another allele 2 (263 bp) [38],* SNCA* gene expression is increased in carriers of the* SNCA* D4S3481 allele 1 (261 bp). Our data showed a seven-year delay in the disease onset in carriers with two allele 1 of* SNCA* D4S3481 (Table 3). While the biological function of* SNCA* rs894278 G allele remains to be determined, the* SNCA* rs11931074 allele T is associated with reduced serum *α*-synuclein [39], even though it is actually located distal to the 3′UTR sequence. Due to the weak linkage disequilibrium of* SNCA* rs11931074 and rs894278, it indicates that the* SNCA* rs894278 GG genotype may also reduce* SNCA* gene expression. Our data showed that* SNCA* rs894278 GG genotype carriers have an earlier PD onset by three years on average (Table 3). In summary, our combined genetic data indicated the expression levels of* SNCA* play an important role at the onset age of PD with lower* SNCA* expression associated with earlier onset and the higher* SNCA* expression associated with older PD onset.

In PD, different PD susceptibility genes occur in early onset compared with late onset of PD [40, 41], and the* MAPT* gene did not independently influence the age of PD onset [13]. Although *α*-synuclein fibrillisation and Lewy body formation in human brain are the key and essential pathogenic process in PD, substantial loss of dopaminergic neurons is more likely responsible for the onset of the clinical motor symptoms diagnostic of PD. Recent evidence suggests *α*-synuclein is a critical protein in dopaminergic neuron survival. During normal ageing, increased* SNCA* expression in the brain has been observed in both healthy humans and monkeys [42]. Interestingly, increased* SNCA* expression is associated with an increased lifespan in transgenic* C. elegans* [43] and* SNCA* variants are associated with an increase in human lifespan [44]. These data may suggest that a reduction in biologically functional *α*-synuclein, whether through aggregation or reduced expression, could precipitate the neurodegeneration in PD [45, 46].

The merit of this study is the interrogation of two populations independently, with comparable results in both cohorts. Our data suggest that different therapeutic strategies should be considered based on polymorphisms in the* SNCA* gene of individual patients and that maintaining a certain level of biologically functional *α*-synuclein is an important consideration in targeting *α*-synuclein for therapies [44, 47]. Our results emphasize that a better understanding of genome-wide risk factors on the clinical quantitative traits in patient with PD, that is, age at onset and severity of motor and nonmotor symptoms, may assist with future personalised medicine for PD.

## Supplementary Material

The primers sequences and genotyping methods used in this manuscript are provided in the supplementary table 1. Direct sequencing method was used for genotyping rs894278 and rs3744456, and restriction fragment length polymorphism (RFLP) method was used for genotyping rs11931074, rs242557 and rs17650901. Capillary electrophoresis method was used for D4S3481 marker genotyping.

## Figures and Tables

**Table 1:** Subjects demographic information.

PD cohorts	*N*	Female : male	Ethnic origin	Age (y/o) (mean ± SD)^*^	Onset (mean ± SD)
Australians	123	57 : 66	Caucasian	68 ± 9	60 ± 11
Chinese	289	119 : 170	Han	63 ± 9	58 ± 10

*N*: number; y/o: years old; SD: standard deviation; ^∗^ *P* < 0.001.

**Table 2:** *SNCA* but not *MAPT* gene associates with age of PD onset (Random-Effects Models).

Cohorts	SNPs	Genetic Inheritance Model	Number	*F* value	*P* value
Australians (123 cases)	*SNCA * D4S3481	Genotypes (00, 01, 02, 11, 12, 22)	14, 34, 6, 51, 15, 3	3.953	0.002
		Allele 0 carrier status (2, 1, 0)	14, 40, 69	5.606	0.005
		Dominant (2 + 1, 0)	54, 69	11.291	**0.001**
		Recessive (2, 1 + 0)	14, 109	1.996	0.160
		Allele 1 carrier status (2, 1, 0)	51, 49, 23	8.408	**<0.001**
		Dominant (2 + 1, 0)	100, 23	8.742	0.004
		Recessive (2, 1 + 0)	51, 72	13.840	**<0.001**
		Allele 2 carrier status (2, 1, 0)	3, 21, 99	1.966	0.145
		^*^Dominant (2 + 1, 0)	24, 99	1.853	0.176
	*MAPT* ^*^rs17650901	Dominant (GG + AG versus AA)	86 versus 37	3.272	0.073

Chinese (289 cases)	*SNCA *				
	rs11931074	Dominant (GG + GT versus TT)	172 versus 117	0.638	0.425
		Recessive (GG versus GT + TT)	46 versus 243	0.358	0.550
		Additive (GG versus GT versus TT)	46 versus 126 versus 117	0.374	0.689
	rs894278	Dominant (GG + GT versus TT)	182 versus 107	0.665	0.415
		Recessive (GG versus GT + TT)	47 versus 242	5.20	**0.023**
		Additive (GG versus GT versus TT)	47 versus 135 versus 107	2.592	0.077
	*MAPT *				
	rs2425577	Dominant (GG + GA versus AA)	190 versus 99	0.583	0.446
		Recessive (GG versus GA + AA)	55 versus 234	0.026	0.871
		Additive (GG versus GA versus AA)	55 versus 135 versus 99	0.297	0.744
	^*^rs3744456	Dominant (CC + CG versus GG)	77 versus 212	1.574	0.211

^∗^Only dominant inheriting pattern is adopted due to rare minor allele frequency of the homozygote.

**Table 3:** Association between *SNCA* and age of onset of Parkinson's disease after adjusting for gender.

Cohorts	Polymorphism	*N*	Genetic Inheritance Model	Genotype	*N*	Age at onset (s.e.)	*P* value
Australians	*SNCA* D4S3481	123	Recessive	No allele 1-one allele 1	72	57 (12)	0.002
				Two allele 1	51	64 (8)	

Chinese	*SNCA* rs894278	289	Recessive	G/G	47	55 (2)	0.015
				T/T-G/T	242	58 (1)	

*N* = number; s.e. = standard error.
