# Supplementary material for: SNCA Gene, but Not MAPT, Influences Onset Age of Parkinson's Disease in Chinese and Australians
Source: Biomed Res Int. 2015 Apr 15;2015:135674. doi: 10.1155/2015/135674 (PMC4413514; doi:10.1155/2015/135674)
Supplement: Supplementary file 1 — The primers sequences and genotyping methods used in this manuscript are provided in the supplementary table 1. Direct sequencing method was used for genotyping rs894278 and rs3744456, and restriction fragment length polymorphism (RFLP) method was used for genotyping rs11931074, rs242557 and rs17650901. Capillary electrophoresis method was used for D4S3481 marker genotyping. [file 135674.f1.pdf]

Independent and joint effects of the *MAPT* and *SNCA* genes on onset age of Parkinson's disease

Yue Huang[1*], Gang Wang[2*], Dominic Rowe[3], Ying Wang[2], John BJ Kwok[1], Qin Xiao[2], Frank Mastaglia [4], Jun Liu[2], Sheng-Di Chen[2#], Glenda Halliday[1#]

Supplementary Table 1 Primers Sequences and Genotyping Methods

Gene	SNP	Chromosome position	Primers	Genotyping methods	PCR products (bp)	RFLP Products (bp)
SNCA	D4S3481	~10 kb upstream	F: CCTGGCATATTTGATTGCAA<br>R: GACTGGCCCAAGATTAACCA	Capillary electrophoresis on an ABI 3100 Genetic Analyzer (Applied Biosystems)	259, 261, 263	NA
	rs11931074	90639515	F: ACCTATCTATTCCGCCCATCC<br>R: TAGCCAAATCTATAAGAGCAACAC	BsrI (invitrogen)	351	T allele: 351<br>G allele: 203, 148
	rs894278	90734535	F: GACGGAAGTGAAGGCAGTG<br>R: GGAAGGAGGGCAGAGTTTT	Direct DNA sequencing (3730xl DNA analyser, Applied Biosystems, Foster City, CA, USA)	399	NA
MAPT	rs3744456	43972176	F: GGTGCGTCTTCCAGGGGACA<br>R: GCGAATCTGGTGGTGAAGAC	Direct DNA sequencing (3730xl DNA analyser, Applied Biosystems, Foster City, CA, USA)	1247	NA
	rs242557	44019712	F: GACACTAATAAGGGAAAATCTC<br>R: GACTGTGGAAGGCTCTGA	APaLI (invitrogen)	405	A allele: 405<br>G allele: 236, 169
	rs17650901	44039691	F: CAACACTCCTCAGAACTTATC<br>R: CAGTGATCTGGGCCTGCTGTG	AluI (New England Biolabs Inc.)	229	A allele: 229<br>G allele: 183, 46

NA=not applicable

Supplementary Figure: No gender difference on the *SNCA* genetic effects of the onset age in PD

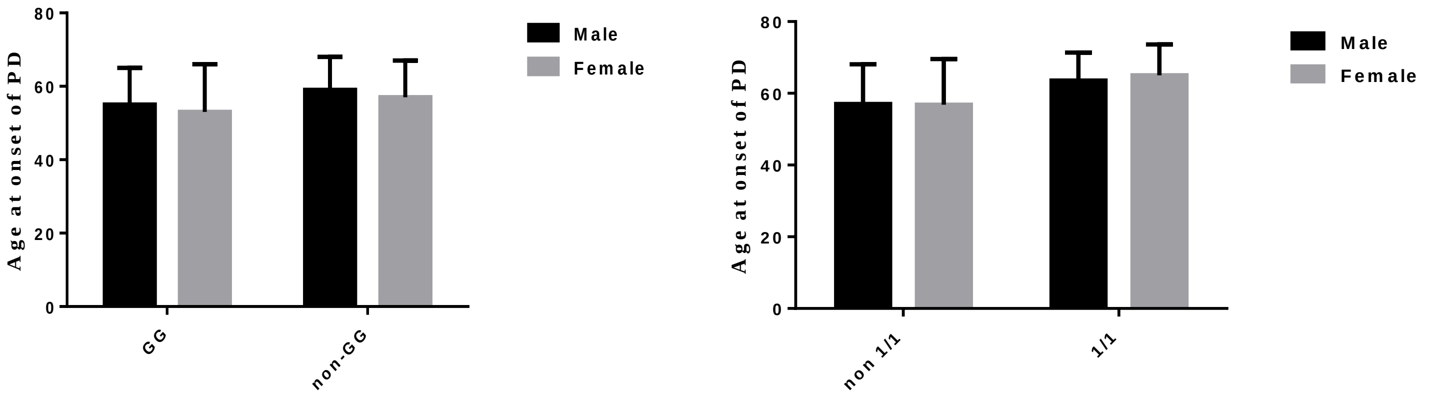

A. *SNCA* rs894278 genotype in Chinese

B. SNCA-Rep1 genotype in Australian
